# Transparent Photocatalytic Thin Films on Flexible Polymer Substrates

**DOI:** 10.3390/ma11101945

**Published:** 2018-10-11

**Authors:** Nives Vodišek, Andraž Šuligoj, Dorota Korte, Urška Lavrenčič Štangar

**Affiliations:** 1 Laboratory for Environmental and Life Sciences, University of Nova Gorica, SI-5001 Nova Gorica, Slovenia; nives.vodisek@gmail.com (N.V.); dorota.korte@ung.si (D.K.); 2 Faculty of Chemistry and Chemical Technology, University of Ljubljana, SI-1000 Ljubljana, Slovenia; andraz.suligoj@fkkt.uni-lj.si; 3 National Institute of Chemistry, SI-1000 Ljubljana, Slovenia

**Keywords:** photocatalysis, self-cleaning surface, TiO_2_, ZrO_2_, SiO_2_, transparent films, thermosensitive substrates, PMMA, PVC, PES, PVDF

## Abstract

Self-cleaning and/or photocatalytic films on polymer substrates have found numerous applications during the past decades. However, the common demand for high-temperature post synthesis treatment limits the application to temperature resistant substrates only. Herein, we prepared self-cleaning photocatalytic films on four thermosensitive polymeric substrates: polyvinyl chloride (PVC), polymethyl methacrylate (PMMA), and acryl coated polyester (PES) fabric (D2) with poly(vinylidene fluoride) (PVDF) containing lacquer (D1). TiO_2_ was prepared via a low-temperature sol-gel process using titanium(IV) isopropoxide and zirconium(IV) butoxide as precursors with various loading levels of Zr; 0, 5, 10, and 20 mol.%, and deposited on the substrates by using a SiO_2_ binder in form of thin films (ca. 200 nm thick) via dip-coating. The films were characterized by SEM, hardness test, UV-Vis, photothermal beam deflection spectroscopy, and IR spectroscopy, while photocatalytic activity was measured by the fluorescence-based method of the terephthalic acid probe and wetting by contact angle measurements. Films containing 10 mol.% of Zr showed the best compromise regarding photocatalytic activity and mechanical stability while from substrates point of view PVC performed the best, followed by PMMA, D1, and D2. The beneficial role of SiO_2_ binder was not only guaranteeing excellent mechanical stability, but also to prevent the D1 polymer from deterioration; the latter was found to be labile to long-term solar-light exposure due to degradation of the top PVDF layer.

## 1. Introduction

Nature-inspired self-cleaning surfaces that are usually either superhydrophobic or superhydrophilic have been getting more and more attention over the years [1,2]. The superhydrophilic effect can be achieved by changing the (nano)structure or chemistry of the surface and can be accomplished via “bottom-up” or “top-down” methods [1]. In the former approach, an active agent is synthesised, which often comprises of nanoparticles, thus functioning not only as a catalyst for cleaning purposes, but also as a component directing the final nanostructure of the material surface. As such, titanium dioxide (TiO_2_) has been reported numerous times as the active component of the self-cleaning films [3,4]. However, TiO_2_ has some limitations since it reduces the total transmittance of the films and the non-hydrophilic water contact angle in the dark is usually quickly re-established. Also, it has a relatively high band gap (3.2 eV for anatase), hence being limited to UV-illumination only. In order to circumvent these limitations, composites with other oxides have been proposed.

ZrO_2_ is known for its high permittivity, semiconducting behaviour, and thermal stability; it can thus be used with titania in photocatalytic applications. Moreover, it has a high coefficient of expansion and thus results in a reduced formation of cracks during annealing. It was reported that coupling those two semiconductors can prevent anatase to rutile phase transformation of TiO_2_ while calcination [5,6,7] and by increasing the surface area and forming new surface sites, it can additionally improve photocatalytic activity. Also, the incorporation of ZrO_2_ in TiO_2_ improves mechanical stability and decreased the ageing effect of TiO_2_ [8].

On the other hand, SiO_2_ can be applied as a protective layer for a soda-lime glass to prevent harmful migration of Na^+^ from the substrate into the TiO_2_ layer during the high-temperature heat treatment [9]. SiO_2_ has also been reported to pronounce the hydrophilicity of films, which can be maintained even in the dark [4,10,11]. Last but not least, SiO_2_ acts as a binding component, improving adherence to the substrate, mainly glass [8], and also acts as a dispersing agent for TiO_2_ nanoparticles [12].

Photocatalytic thin films have found applications on various surfaces, for example, glass, plastics [13,14,15], textiles [16,17,18,19,20,21,22,23,24,25], concrete [26,27,28], tiles [29,30], facade [28,31,32], mirrors [26,33,34], polymer membranes [35,36], etc. Polymeric substrates are challenging for various reasons: (i) they are commonly thermosensitive; (ii) proper adhesion of the films and their long-term stability are hard to achieve; (iii) homogeneity, transparency, and unchanged optical properties of substrates is also difficult to achieve; and very importantly, (iv) photo-yellowing and photodegradation of organic substrate is commonly observed in such systems. We chose the dip-coating procedure for thin films deposition, which demands a preparation of proper solution with suitable rheological properties. In the literature, we came across different approaches to prepare polymeric substrates, use of various surface pre-treatment method [37,38], or use of special deposition technique [39,40,41,42]; in other cases, various additives were used and binders to provide a better adhesion of the film [43,44]. SiO_2_ is often chosen as a protective layer in such substrates [24,45,46] for means of preventing the photodegradation of the substrate.

Herein, zirconia and silica were incorporated into the titania film structure to study their multifaceted influence, following the successful application of such a sol system on glass substrates previously reported [8]. The films were prepared via low-temperature procedure and it can be applied to many substrates because such films do not demand high-temperature post-treatment. The substrates of choice were polyvinyl chloride (PVC), polymethyl methacrylate (PMMA), and highly studied poly(vinylidene fluoride) (PVDF) coating. The photocatalytic activities were thoroughly investigated by contact angle measurements and by following the oxidation of terephthalic acid; additionally, the long-term stability of the films and a prevention strategy against photo-yellowing of the polymers, which involves the incorporation of SiO_2_ is finally discussed.

## 2. Materials and Methods

### 2.1. Materials

Chemicals that we used in this study are: tetraethyl orthosilicate (TEOS, 98%) from *Acros Organics* (Geel, Belgium); titanium(IV) isopropoxide (TTIP, 98%), zirconium(IV) butoxide (ZTB, 80 wt %) from *Aldrich* (Steinheim, Germany); terephthalic acid (TPA) from *Alfa Aesar* (Karlsruhe, Germany), absolute ethanol, hydrochloric acid (37%), 2-propanol from *Carlo Erba* (Val de Reuil, France); hydroxyethyl-cellulose (HEC 2 wt %) from *Fluka*; ethanol (96%) from *Itrij* (Radovljica, Slovenia); Levasil 200/30% from *Obermeier* (Bad-Berleburg, Germany); perchloric acid (70%), 2-propoxyethanol, 1-propanol, and Resazurin and Glycerol (99.5%) from *Sigma-Aldrich* (Steinheim, Germany), and NaOH from *VWR* (Wien, Austria).

### 2.2. Preparation of the Materials

The TiO_2_ sol was prepared through sol-gel reflux processing, as previously described [8] by dissolving TTIP, absolute ethanol, and ZTB; as a peptizing agent, an aqueous solution of HClO_4_ was used. Molar percentage ratio of Zr/Ti was varied by varying the amount of zirconium(IV) butoxide at fixed TTIP. As a result, a series of samples were prepared with Zr/Ti molar ratio of 5%, 10%, and 20%. The obtained samples were designated as Ti5Zr, Ti10Zr, and Ti20Zr, respectively. Pure TiO_2_ was also synthesized by the same procedure, without adding the ZTB and it was designated as Ti0Zr. SiO_2_ binder solution was prepared from TEOS, colloidal SiO_2_ (Levasil 200/30%), HCl, and 2-propanol. 1-propanol and 2-propoxyethanol were finally added to produce a solution with suitable rheological properties [8]. TiO_2_ and SiO_2_ sols were combined and mixed thoroughly overnight. This sol was then deposited by the dip-coating technique with immersion and withdrawal speed of 100 mm/min on four different polymer substrates: PVC foil, PMMA, D1, and D2. Composition and thermal stability of the substrates are given in Table 1. After dip-coating, the samples were dried first with a blow-dryer and then with heat-gun (Black&Decker) for 30 s on each side from ca. 30 cm distance. On every substrate, the coating-heating cycle was repeated to obtain three layers of each sample.

### 2.3. Characterization

Specific surface area was determined according to multi-point Brunauer-Emmett-Teller theory (BET) and thermal analyses were conducted on thermogravimeter and differential scanning calorimeter (TGA-DSC) on analogous powder samples. These results are presented in our previous research paper [8]. Fourier-transform infrared (FTIR) spectra from 4000 to 400 cm^−1^ were obtained by Perkin Elmer 100 with GladiATR™ Single Reflection ATR Accessory (PIKE Technologies, Madison, WI, USA), X-ray powder diffraction (XRD) patterns were obtained with RIGAKU MiniFlex 600 (Rigaku, Tokyo, Japan) with the copper source, providing X-rays of the wavelength of 1.54 Å. The diffraction data were collected in the 2Θ range of 20–80°. From XRD data, size of nanocrystalline anatase particles was calculated by the Scherrer equation. UV-Vis transmittance of the thin films and diffuse reflectance spectroscopy (DRS) measurements of the powder analogues were performed on Lambda 650 Perkin Elmer (PerkinElmer, Waltham, MA, USA) in the range of 200 to 800 nm. The powder samples for XRD and UV-Vis DRS measurements were derived from the corresponding deposits of the solutions after heat treatment at 150 °C. Additionally, band gap values of the semiconducting thin films were determined by the photothermal technique [39,40] that is Beam Deflection Spectroscopy (BDS) [47].

**Figure 1:**
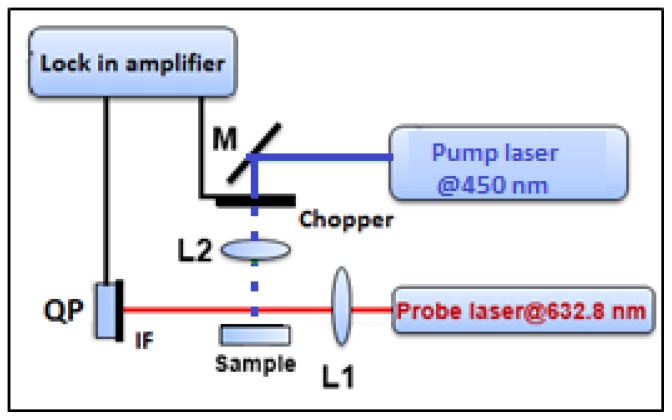
Schematic of the beam deflection spectroscopy (BDS) experimental setup. L1, L2: lenses, M: reflecting mirrors, IF: interference filter, QP: quadrant photodiode.

The BDS technique was performed using the lab-built experimental setup that is presented in Figure 1. The sample is illuminated by an intensity modulated light beam (pump beam) [47] from a solid state laser (Coherent, OBIS 455) with 450 nm and 50 mW of output wavelength and power, respectively. It is modulated by mechanical chopper (SCITEC INSTRUMENTS, Control unit model 300C, chopping head model 300CD, chopping disks model 300H, Scitec Instruments Ltd, Wiltshire, UK), and heats the sample perpendicularly to its surface. The sample is placed on a three-dimensional (3D) translation stage (CVI, Model 2480M/2488) to vary its position in *x*, *y* and *z*-direction and to optimize the experimental configuration. The absorbed energy induces temperature oscillations (TOs) not only in the sample but also in its surroundings. The TOs are probed by He-Ne laser beam (probe beam) of 632.8 nm output wavelength and 2 mW output power (Model 25-LGR-393-230, CVI MELLES GRIOT, Pittsfield, MA, USA). The interaction of probe beam (PB) with TOs results in PB intensity change that is detected by a quadrant photodiode (Model C30846E, RBM–R. Braumann GmbH, Langenbach, Germany) equipped with an interference filter (Edmund Optics) and connected to the lock-in amplifier (Stanford research instruments, Model SR830 DSP).

Scanning electron microscopy (SEM,) images were obtained on a Zeiss Supra 35 VP microscope (Carl Zeiss NTS GmbH, Oberkochen, Germany) operating at 1 kV. The samples were pre-coated with carbon (ca. 8 nm).

The mechanical scratch resistance of the films was determined by a pencil hardness test (ISO15184:1998) using Elcometer 501 (Elcometer Instruments, Manchester, United Kingdom). ATR FTIR spectra from 4000 to 400 cm^−1^ with the resolution of 4 cm^−1^ were obtained by Perkin Elmer 100 with GladiATR™ Single Reflection ATR Accessory. Samples during contact angle measurements and resazurin test were irradiated in a UV chamber set at λ_max_ = 365 nm and 2.3 mW/cm^2^ of power. Suntest XLS+ from Atlas, USA, was used for the prolonged irradiation of D1 sample (Xenon light, daylight filter, irradiation intensity 750 W/m^2^ (300–800 nm), with UVA fraction of 0.62 W/m^2^ (300–400 nm)) to determine the possible damage upon ageing. Contact angle measurements were carried out on goniometer CAM 100 from KSV instruments (KSV Instruments, Finland).

### 2.4. Photocatalytic Activity

Photocatalytic activity was determined by the fluorescence-based method with terephthalic acid [8,48]. A mixture containing terephthalic acid, an aqueous solution of NaOH and ethanol-based HEC solution was deposited on the catalyst surface with the dip-coating technique (withdrawal speed 10 cm/min). Before the deposition, samples were irradiated for one hour (λ_max_ = 365 nm, 2.3 mW/cm^2^). After coating with TPA, they were placed back in the UVA chamber. During illumination, samples for analyses were obtained by periodically washing off the TPA layer with a mixture of ethanol/water solution (0.25 mL/cm^2^). Fluorescence in the collected samples—due to the formation of hydroxyterephthalic acid (HTPA), a hydroxyl radical product of TPA—was analyzed by using microtiter plate reader spectrofluorometer (Infinite F200 Microplate Reader, Tecan, Männedorf, Switzerland), with an excitation wavelength 320 nm and emission wavelength 430 nm.

The activity of thin films was also visually tested with resazurin (Rz) dye test. The Rz solution was prepared by dissolving Rz dye (250 mg) in deionized water (50 mL) and mixed overnight to completely dissolve the dye. A sacrificial agent (e.g., glycerol) for the photogenerated holes was not used, as we wanted to test the wetting of the surface also. Hence, generated holes react with water (or surface –OH) and prevent electron-hole recombination. A drop (0.5 mL) of such prepared Rz ink was put on the surfaces. Samples were then put in the UVA chamber and irradiated by UV light; photos were taken in 15 min intervals for 2 h to observe the kinetics.

## 3. Results and Discussion

### 3.1. Materials Characterization

**Figure 2:**
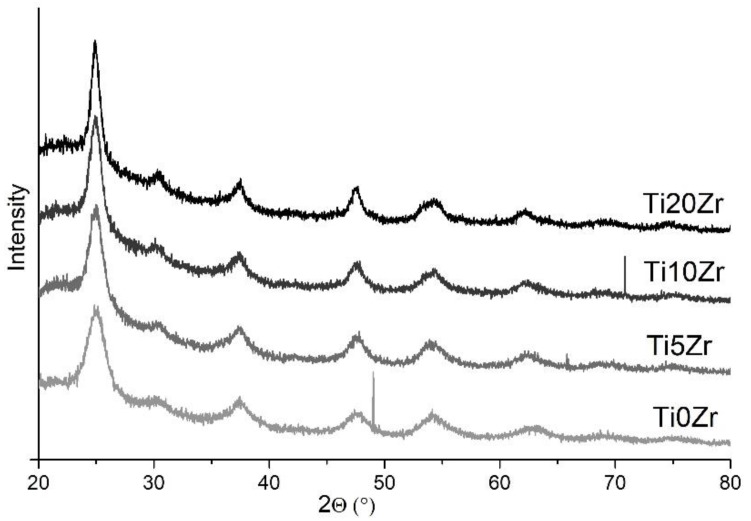
X-ray powder diffraction (XRD) powder patterns.

X-ray powder diffraction (XRD) patterns of the samples with various Zr content are presented in Figure 2. In all samples diffraction peaks at 25°, 38°, 48°, 54°, and 63° are observed despite low-temperature preparation route and are assigned to anatase reflection planes (101), (004), (200), (105), and (204), respectively [49,50]. Less intense diffraction peak at around 30° is ascribed to brookite TiO_2_ phase reflection plane (121) [51], which is present as a minor crystalline phase. As expected, the presence of zirconia and silica is not reflected in the diffraction patterns due to the amorphous character of these phases. XRD patterns showed that with higher loading of Zr, diffractions are sharper, which also indicates the increase of the anatase crystallite size. Particle sizes from XRD patterns were calculated by using the Scherrer equation based on the full width at the half maximum (FWHM) of the (101) diffraction peak. Thus calculated sizes are presented in Table 2.

**Figure 3:**
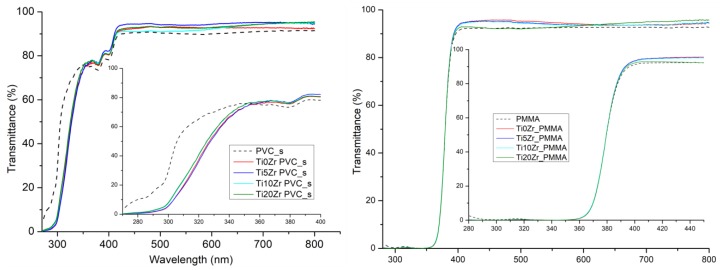
UV-Vis transmittance spectra for polyvinyl chloride (PVC_s) (**left**) and polymethyl methacrylate (PMMA) (**right**) samples.

In Figure 3 UV-Vis transmittance spectra of the films on PVC_s and PMMA are shown, omitting the D1 and D2 substrates since they are not transparent. The coated samples show a higher transmittance than bare substrates in the visible region; the reason being the presence of silica, which has an antireflective property [52,53,54,55]. As the PMMA substrate does not transmit the UV light below 360 nm, no major difference among them is observed in the UV region. On the other hand, the PVC_s substrate transmits a significant portion of the UV light, hence differences can be seen. The substrate only starts to strongly absorb at λ < 320 nm but upon deposition of the photocatalyst thin films the transmittance margin of the samples shifts to higher wavelengths i.e., 350 nm (inset in Figure 3), which is close to the absorption edge of TiO_2_.

For this reason, we determined the band gap energies of the samples. They were obtained by two different methods, i.e., UV-Vis diffuse reflectance using powders and with laser beam deflection spectroscopy (BDS) using thin films [47]. Regarding the former method, one can determine the indirect band gap (Kubelka-Munk model) from the plot (F(R)*hν)^1/2^ vs. hν, where F(R) = (1 − R_∞_)^2^/2R_∞_ [56]. Values of the band gap are decreasing with the increased size of the crystallites (Table 2).

**Figure 4:**
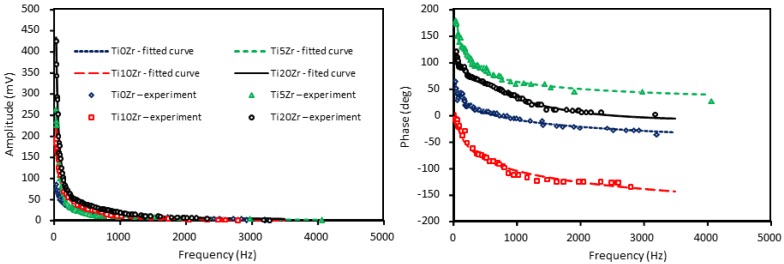
The amplitude (**left**) and phase of BDS signal (**right**) for unmodified and Zr-modified composite samples. Points represent the experimental data whereas continuous lines the best fitting using model presented in [47].

As regarding the BDS band gap measurements, the amplitude and phase of the signal are collected as a function of the modulation frequency of the pump beam for the unmodified and Ti*X*Zr composite samples (Figure 4).

It is seen that the signal falls off exponentially with the increase in the modulation frequency of the TOs. Thus, to optimize the experimental conditions the probe beam is tightly focused and carefully aligned close to the sample surface, just to skim it. For that reason, the measurement is performed on flat samples with small lateral dimensions. Furthermore, both the amplitude and phase of the BDS signal are sensitive to optical properties (energy band gap) of the examined samples, what allows for determining its value on the basis of the least-squares method of fitting the theoretical curve to the experimental data [47]. The best theoretical fitting to the experimental data is obtained for the band gap values presented in Table 2. The variation in the value of the energy band gap that was caused by the introduction of ZrO_2_ into the TiO_2_ material is rather low (approximately 5%) and it can be attributed to the effective value of the whole composite and its changes in composition.

Comparing the two methods, the values match very well and the trend is the same while using both methods. With higher loadings of Zr, the band gap energy is decreasing, which is inversely correlating with the anatase particle size (Table 2); Ti0Zr has the smallest and Ti20Zr the biggest particles. Moreover, it is also well agreed with the data reported elsewhere [57] and it can be described by the quantum size effect.

**Figure 5:**
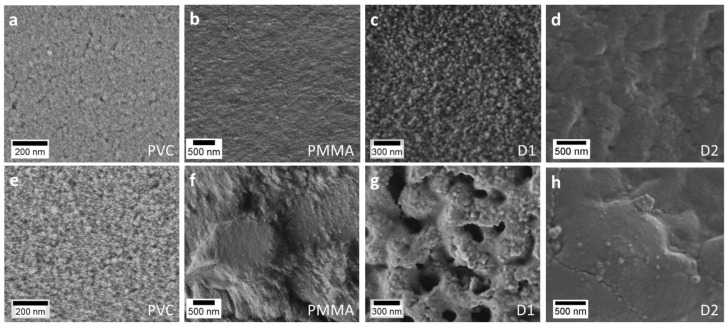
Typical scanning electron microscopy (SEM) images of Ti0Zr film (**a**–**d**) and Ti10Zr film (**e**–**h**) on four different substrates: PVC_s (**a**,**e**), PMMA (**b**,**f**), D1 (**c**,**g**), and D2 (**d**,**h**).

In Figure 5 typical SEM images of the Ti0Zr and Ti10Zr thin films on PVC_s, D1, D2, and PMMA substrates are presented. The growth of the particles size and a trend towards better-resolved shapes can be seen in the 0 to 10% Zr sample series. Also, the structure of the films is clearly rougher upon Zr introduction, which is in agreement with our previous publication with such films deposited on glass substrates [8].

**Figure 6:**
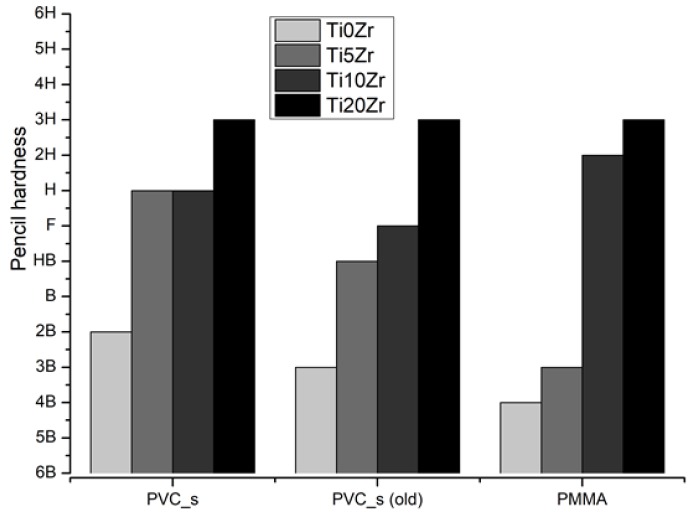
Mechanical stability measured by hardness pencil test.

Mechanical stability tests were not possible to be conducted on substrates D1 and D2, because of the too rough texture of these substrates (PES fabric inside). Mechanical stability of PVC_s and PMMA specimen (Figure 6) show that the highest stability was obtained on the sample with the highest Zr content on both substrates, while sample without Zr was the least mechanically stable. Interestingly, although the trend of higher scratch resistance with higher Zr concentrations is present in both substrates, lower Zr loadings (0 and 5%) performed better on PVC_s substrate, while 10% loading shows higher stability with PMMA substrate relative to PVC_s. We also performed a scratch test on one-year-old PVC_s films stored in the dark (Figure 6). The Ti20Zr retained the stability, but the other three films lost their mechanical stability for one or two pencil hardness, which is in agreement with the measurements on glass substrates [8].

**Figure 7:**
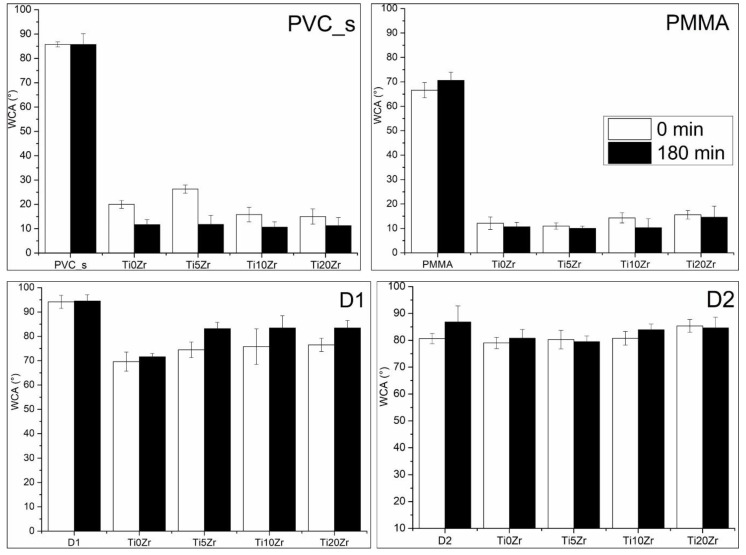
Water contact angle values for films with various Zr concentration deposited on different substrates, as marked by their first columns. Error bars represent standard error of the mean (N = 5).

Surface properties were evaluated by measuring water contact angles (WCA) and they are shown in Figure 7. The WCAs of bare substrates were approximately 85° except for PMMA (65°) and D1 (95°); the high WCA of the latter is the consequence of the presence of fluorine in the PVDF top layer, which is not present in the D2 neat substrate. The WCAs of all bare substrates do not show significant change after 3h of UV irradiation. On the other hand, coating the surfaces with TiO_2_ films showed much lower initial WCAs in PVC_s (15–25°) and PMMA (15–20°) substrates; it did not, however, affect the D1 and D2 initial WCAs—they remained relatively high, i.e., 70° and 80° for D2 and D1 substrate, respectively. Upon irradiation, the WCAs decreased in PVC_s and PMMA substrates, although no clear correlation can be deduced regarding the concentration of Zr in the TiO_2_ film. Interestingly, the WCAs of films on D1 and D2 substrates actually increased upon irradiation, although mostly not in a statistically significant manner. From these observations, we can conclude that the films are highly active on PVC_s and PMMA substrates but not on acryl coated PES substrates. However, hydrophilicity might not be the most important measure related to the photocatalytic activity for surfaces [58] due to their high dependence on surface charge and roughness, i.e., D1 and D2 exhibited millimetre-sized textural features, hence the determination of WCAs was difficult and varied greatly due to such surface effects.

### 3.2. Photocatalytic Activities

**Figure 8:**
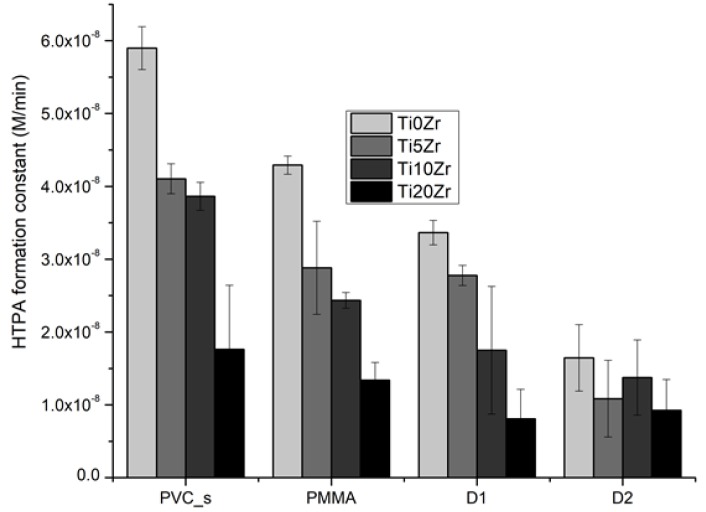
Photocatalytic activities determined by the formation constant of hydroxyterephthalic acid. Error bars represent standard error of the mean (N = 3; three different spots on the sample, as suggested in ref. [48]).

To determine photocatalytic activities, we conducted measurements of the HTPA formation rate on films (Figure 8). Generally, the addition of zirconium decreased the photoactivity of the films, regardless of the substrate used. This is in accordance with our previous study on glass substrates [8] and the reason for such a trend can be attributed to larger crystal size and consequently lower surface area, together with an increasing portion of inactive zirconia phase. Interestingly, the lowering of the activity with 20% Zr addition is similar in all of the substrates used (~70% decrease), except for D2 substrate where activity was lowered for only 44% (not statistically significant). However, this sample also showed the lowest activity in general. The reasons for this are still unclear.

**Figure 9:**
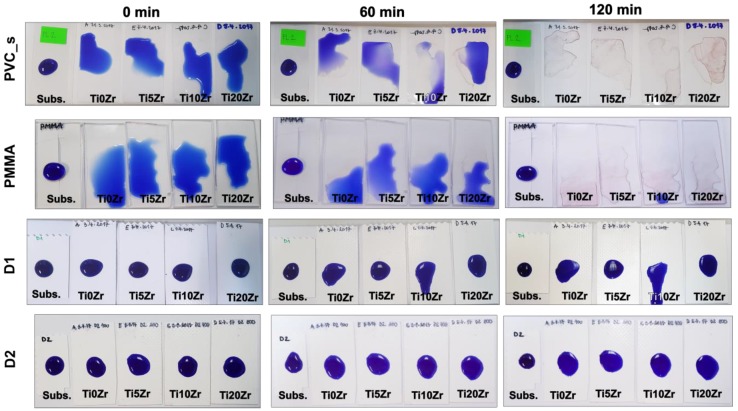
Degradation of the resazurin dye under UV light for substrates PVC (**a**), PMMA (**b**), D1 (**c**), and D2 (**d**).

The third and more visible-oriented photocatalytic test was the reduction of resazurin dye on the surface of the materials (Figure 9). Clearly, the neat substrates showed no discolouration of the dye, even though the irradiation source was UV-light. The wetting of the surface with increasing irradiation times was observable in substrates PVC_s and PMMA, while in D1 and D2 substrates, this effect was not seen, thus confirming their less hydrophilic nature when compared to PMMA and PVC_s. Among the latter two, PMMA is showing faster wetting of the surface with the resazurin, which is in accordance with the WCAs of these substrates, i.e., PMMA showed more hydrophilic nature. Regarding the reduction of resazurin, only PVC_s and PMMA substrates showed a remarkable reduction of resazurin to resorufin (pink) and even further to dihydroresorufin (colorless), as shown in the Figure 9. PMMA, however, showed slightly higher discolouration at final irradiation times (Figure S1). Generally, a higher percentage of Zr produced smaller discolouration in PVC_s and PMMA substrates, while D1 and D2 samples were less active, and such a trend was not observed. D2 substrate showed a measurable blue colour change, while this was not the case in the D1 substrate. We can conclude that the TiO_2_/SiO_2_ films on PMMA and PVC_s substrates were more successful in photocatalytic degradation; they were more active in TPA oxidation and resazurin reduction as compared to the D1 and D2 substrates.

### 3.3. Photostabilities of the Materials

The instability of acrylic coatings in the presence of TiO_2_ and UV-irradiation has been described in the literature [59]. The resistance of PVDF membranes has also been investigated, especially for application in electronic devices [60]. A degradation of the chosen polymeric substrates could be expected due to the formation of the highly reactive radicals or h^+^ themselves by the photocatalyst layer, as confirmed by the HTPA test.

**Figure 10:**
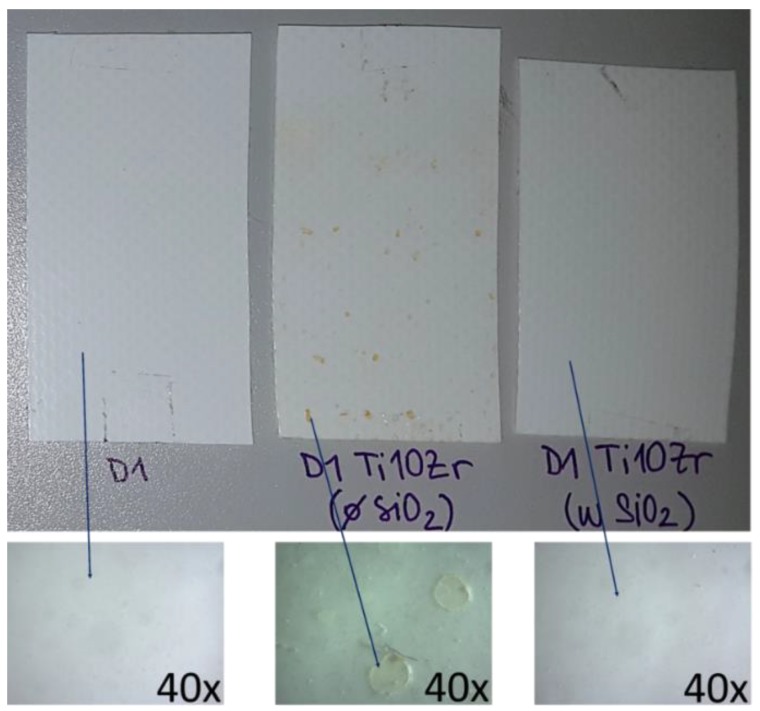
D1 samples exposed to ageing in Suntest chamber with a daylight filter for 14 days. **Left**: a neat substrate; **middle**: Ti10Zr film without SiO_2_ binder; **right**: Ti10Zr with SiO_2_ and their appearance under higher magnification (**below**).

Hence, we performed a simulated ageing experiment; the bare D1 substrate, a D1 substrate with the Ti10Zr film without binder, and a D1 substrate with the Ti10Zr film with SiO_2_ binder were irradiated for 14 days in a Suntest chamber with a daylight filter applied. Figure 10 shows that the neat substrate (stable under UVA/B irradiance) and film with SiO_2_ binder were stable after such prolonged exposure to light. However, pale brown spots appeared on the sample with the photocatalytic layer, lacking the SiO_2_ binder (Figure 10, middle). A closer look (Figure 10, below) showed crater-like surface structures on the substrate surface, which additionally caused part of the photocatalytic layer to detach at such spots.

**Figure 11:**
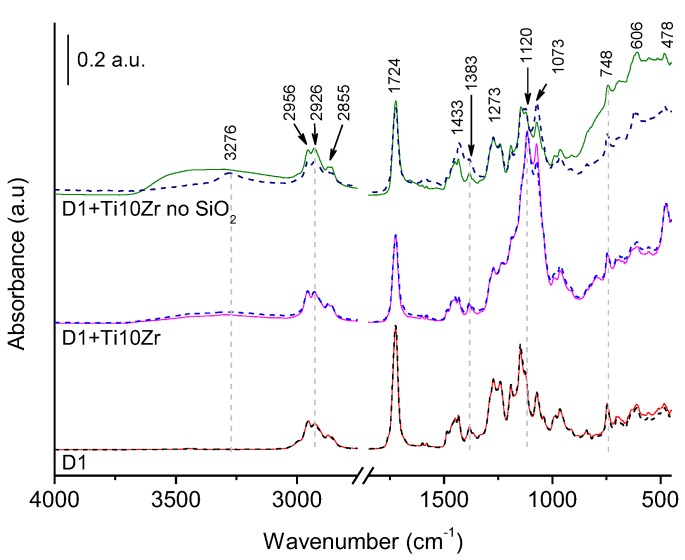
ATR-FTIR spectra of fresh (solid lines) and samples irradiated in Suntest for 14 days (dashed lines).

Further identification of changes in the samples was conducted by attenuated total reflection (ATR) FTIR spectroscopy (Figure 11). The semi-crystalline polymer which sits on top of the acryl-painted PES fabric shows a complex structure and can present several distinct crystalline phases that are related to different chain conformations; commonly α-, β-, and γ-phase (Figure S2). The β-phase is characterized by a peak at 1273 cm^−1^ [61], while α-phase was detected at 612, 967, and 1383 cm^−1^. The presence of γ-phase was also evidenced by the peak at 1240 cm^−1^, thus confirming the presence of a mixture of α-, β-, and γ-phase PVDF on the surface of the polymer support, as stated by the producer [62].

The TiO_2_-coated samples show ν(OH) at 3600–3200 cm^−1^, indicating on a more hydroxylated surface, which is in accordance with the CA measurements. It can be observed, however, that the TiO_2_ films without the presence of SiO_2_ (Ti10Zr no SiO_2_) showed even more hydroxylated surface versus Ti10Zr that contains SiO_2_ binder. The presence of SiO_2_ in the film is additionally confirmed by ν(SiO) at 1120 cm^−1^ and δ(OSiO) at 480 cm^−1^, while the wide absorption around 700–400 cm^−1^ is ascribed to δ(OTiO), due to the presence of TiO_2_ in the coating layer. Films on other substrates have also been characterised in the same way (Figure S3). They all show the presence of δ(OTiO) at ca. 550 cm^−1^ in their structure together with ν(SiO) at 1120 cm^−1^.

Upon simulated ageing, several features in the spectra of TiO_2_ coated D1 sample (without SiO_2_ binder) appeared, i.e., in the sample that exhibited photo-yellowing. In FTIR spectra pointed at the pale brown spot a reduction of absorption bands in the 900–400 cm^−1^ range—vibrations of TiO_2_ lattice—is seen; it indicates the TiO_2_ active layer was masked, probably by organics from the D1 polymer. Also, the 1073 cm^−1^ peak increased, which could be ascribed to ν(CF) [63,64], suggesting the higher exposure of the C–F bonds. The peak at 1383 cm^−1^ also increased together with 1431 cm^−1^ band; the latter belongs to α-phase of PVDF [61]. Indeed, the α-phase shows a more open structure (Figure S2), thus rendering the detection of C–F in a higher number. Second, an increase in the peak at 3266 cm^−1^ was seen together with a decrease of the ν(OH) band. Absorptions at such high frequencies are characteristic for OH, FH, and NH stretching vibrations [64]. Nasef et al. [60] claimed that the mechanism upon electron beam irradiation-mediated degradation of PVDF membrane involved the elimination of HF upon crosslinking. This explains the presence of ν(FH) in the aged films as HF becomes trapped in-between the PVDF and TiO_2_ layer. Additionally, Dzinun et al. studied [65] the prolonged UV-stability of TiO_2_/PVDF dual-layer hollow fibre membranes. Similar observations with respect to the ageing of the PVDF were reported and ascribed to a higher number of carbon-carbon double bonds (–CF=CH–) formed due to the dehydrofluorination, i.e., the elimination of an H–F unit, resulting in the formation of a carbon-carbon double bond. They also reported the appearance of brown colour after prolonged UV-exposure to the consequence of the mentioned phenomenon.

Our data suggest a partially distorted structure of the PVDF polymer and an increase in the α-phase fraction; hence, crosslinking probably appeared to some degree, while the brown colour suggests the formation of double bonds in –(CF=CH)– units. These results corroborate with the WCA measurements, where D1 coated with TiO_2_ layer showed increased hydrophobic nature. That layer included a SiO_2_ binder—this layer (shown in Figure 11), however, also showed a slightly decreased band at 3266 cm^−1^ (ν(OH) band). Hence, the low activity of this substrate can be explained by the decreased concentration of surface –OH species upon irradiation. On the other hand, studies coupling zirconia with titania often suggest on the presence of a heterojunction. The incorporation of Zr in low quantities commonly gives rise to the creation of defects, which indicates the formation of deep energy states below the conduction band [66]. In this study, however, this is not the case, since the concentration of doping was relatively high (5, 10, and 20 mol.%); thus lowering of the band gap was observed due to the increase of the particle sizes. It is thus reasonable to propose a heterojunction formation between ZrO_2_ and TiO_2_. Still, this does not lead to increased photocatalytic activity due to unfavorable band edge positions (see Figure S4). Altogether, Zr probably resided on the surface of titania as oxo-clusters, which were not limiting the growth of anatase crystals, partially shaded its surface, and at the same time provided higher abrasive properties.

However, the presence of SiO_2_ binder inhibited this process, which was proved by the FTIR spectra and photographs (Figure 10) before and after the ageing experiment, and thus it acts beneficially not only at improving the mechanical and optical properties of the films but also extending the stability of the underlying support. Zhou et al. showed [67] the prolonged wettability of TiO_2_ fluorocarbon composite films when SiO_2_ was introduced to the system. The improvement was ascribed to the increased surface acidity due to the presence of Si–O–Ti bonds, which causes a more prominent reaction with surface water molecules. The peak at ~950 cm^−1^, which indicates on this bond was, however, masked in our system by the relatively large absorption of the D1 polymer itself in this region. While this might explain the high activity of the TiO_2_-SiO_2_ system, it does not explain the prolonged UV-resistance. The reasons for such behaviour are under investigation.

## 4. Conclusions

We have demonstrated the applicability of titania-silica-zirconia Ti10Zr sols to the four different polymeric substrates as photocatalytic films for self-cleaning applications. On all of the substrates, we observed self-cleaning property. In general, the transparent PVC and PMMA substrates performed considerably better than acryl coated PES fabrics (D1 and D2) when used as support for low-temperature derived TiO_2_ films. Film on D1 and D2 substrates were also more hydrophobic i.e., possessed lower concentration of surface–OH species, which is the main reason for lower activity and self-cleaning performance than PVC and PMMA. Fast and visible oriented photocatalytic activity test with Rz ink confirmed the TPA results and water contact angle measurements.

The role of silica binder in such sols was not only to guarantee excellent mechanical and optical properties, but also to prevent the D1 polymer deterioration, followed by ageing the materials. The D1 substrate, which had a TiO_2_ layer deposited, was found to be labile to long-term solar-light exposure due to degradation of the PVDF layer on the top of the material. This was prevented with the addition of SiO_2_ in the photocatalytic layer.

The quantification of the photocatalytic activity by means of contact angle measurements was proven difficult and was much better represented employing a faster method of the oxidation of terephthalic acid deposited on the surface by dip-coating. The presence of zirconia in the photocatalyst layer reduced its photocatalytic activity, but, on the other hand, it significantly increased scratch resistance of the coatings.

The tested substrates are used in a wide area of applications, such as aeroplane windshields, canopies, displays, furniture, rear lights, and windshields in vehicles, greenhouses, sigs, tensile structures, etc. We believe self-cleaning films, as presented here, provide an added value to these materials, making maintenance simpler and saving and improving the lifetime of materials.

## Figures and Tables

**Table 1:** Main characteristics of substrates.

Substrate Label	Description
D1	Acryl coated 100% PES fabric (1100 dtex) with PVDF containing lacquer, white, 1 mm thickness. Temperature resistance between −30 and +70 °C. UVA/B stable. Sioen, Belgium.
D2	Acryl coated 100% PES fabric (1100 dtex), white, 1 mm thickness. Temperature resistance between −30 and +70 °C. UVA/B stable. Sioen, Belgium.
PVC_s	PVC foil. Transparent, 0.1 mm thickness, the temperature of glass transition around 80 °C, melting temperature of 100 °C.
PMMA	Polymethyl methacrylate sheet. Transparent, 3 mm thickness, temperature of glass transition around 85 °C, melting temperature around 130 °C.

**Table 2:** Band gap and grain size values.

Sample	Band Gap—Film ^a^	Band Gap—Powder ^b^	Anatase Crystallite Size (101) ^c^
Ti0Zr	3.30 eV	3.23 eV	4.2 nm
Ti5Zr	3.20 eV	3.16 eV	5.0 nm
Ti10Zr	3.15 eV	3.11 eV	5.8 nm
T20Zr	3.05 eV	3.09 eV	7.7 nm

^a^ Determined by BDS; ^b^ Determined by DRS UV-Vis; ^c^ Calculated with the Scherrer equation from XRD data based on the FWHM of the (101) diffraction peak.

## Supplementary Materials

The following are available online at http://www.mdpi.com/1996-1944/11/10/1945/s1, Scheme S1: (a) Photoreduction of resazurin to resorufin and further to dihydroresorufin associated with the color changes. Figure S1: Quantified results of the blue color in the RGB color system for the resazurin dye degradation on different substrates: PVC (a), PMMA (b), D1 (c) and D2 (d). Figure S2: Structures of PVDF models, corresponding to two representative crystalline polymorphs (α- and β-forms). Figure S3: ATR FTIR spectra of photocatalytic thin films on various substrates; PVC (top left), PMMA (top right), D1 (bottom left) and D2 (bottom right). Figure S4: Schematic representation of the band structures of TiO_2_ and ZrO_2_.
