# Supplementary material for: Transparent Photocatalytic Thin Films on Flexible Polymer Substrates
Source: Materials (Basel). 2018 Oct 11;11(10):1945. doi: 10.3390/ma11101945 (PMC6213899; doi:10.3390/ma11101945)
Supplement: Supplementary file 1 [file materials-11-01945-s001.pdf]

Supporting Information for

Transparent Photocatalytic Thin Films on Flexible Polymer Substrates

Nives Vodišek [1], Andraž Šuligoj [2,3], Dorota Korte [1] and Urška Lavrenčič Štangar [2,1,*]
[1] Laboratory for Environmental and Life Sciences, University of Nova Gorica, SI-5001 Nova Gorica, Slovenia
[2] Faculty of Chemistry and Chemical Technology, University of Ljubljana, SI-1000 Ljubljana, Slovenia
[3] National Institute of Chemistry, SI-1000 Ljubljana, Slovenia

1 Quantification of the Resazurin dye degradation

Resazurin dye changes color irreversibly upon UV irradiation at a rate which is proportional with the photocatalytic activity of the underlying film. The color changes are associated with the photoreduction of the resazurin (blue) to resorufin (pink) and subsequently to a further reduced form, dihydroresorufin (colorless). The proposed reaction schemes are illustrated as:

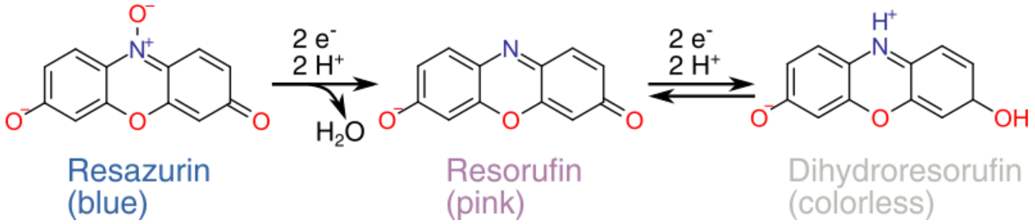

Scheme S1. (a) Photoreduction of resazurin to resorufin and further to dihydroresorufin associated with the color changes.

The relative quantification was done by obtaining the value of the blue color (B) in the RGB color system. The area of interest was the dye drop and if the drop was wetted, a larger area was taken in order to obtain more representative results. The software for image quantification was ImageJ 1.51j8 using the Color Histogram tool.

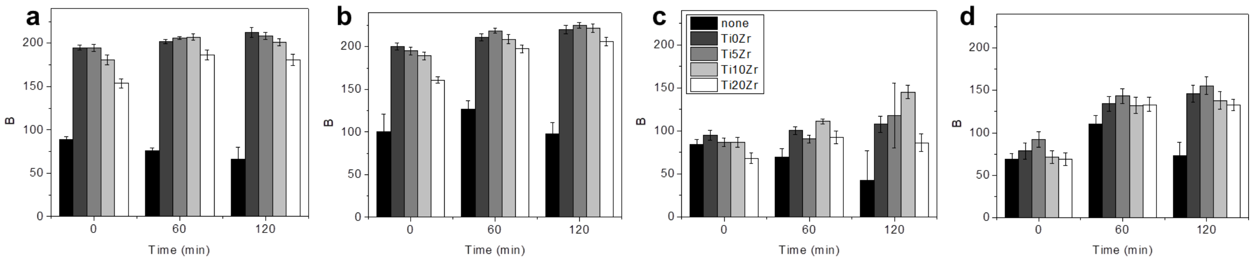

Figure S1. Quantified results of the blue color in the RGB color system for the resazurin dye degradation on different substrates: PVC (a), PMMA (b), D1 (c) and D2 (d). The legend in facet (c) applies to all facets. The blue (B) color ranges from 0 (black) to 225 (light blue), hence the higher the number the lighter the blue color and thus the fastest the degradation/spreading of the colored droplet.

2 Molecular structures of PVDF

The models were drawn in the VESTA 3.4.2 software, by using the optimized α- and β-models of PVDF. The α phase shows a structure which exposes the F-atoms on the surface in greater proportion as compared to the β phase.

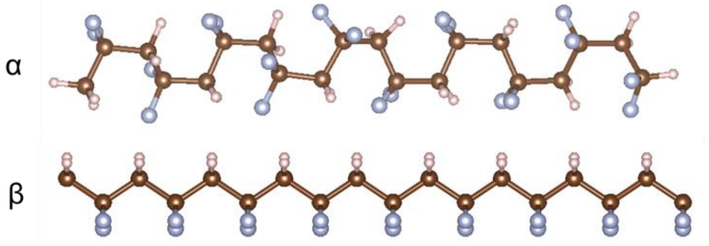

Figure S2. Structures of PVDF models, corresponding to two representative crystalline polymorphs (α- and β-forms). Fluorine, carbon and hydrogen atoms are colored blue, brown and white, respectively.

3 FT-IR spectra of the catalytic films on various supports

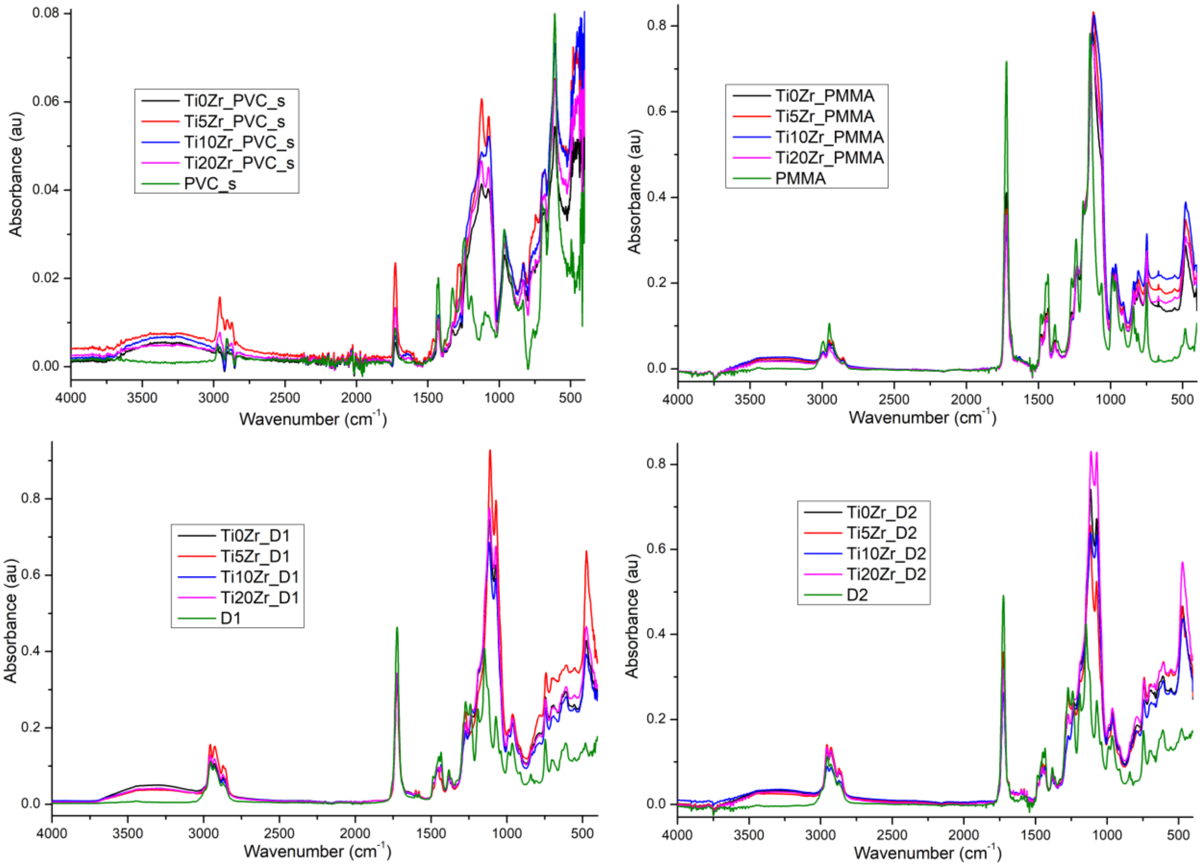

Figure S3. ATR FTIR spectra of photocatalytic thin films on various substrates; PVC (top left), PMMA (top right), D1 (bottom left) and D2 (bottom right).

4 Band Structure

In Figure S4 the schematic diagram of the band positions is shown. Note that the incorporation of Zr^{4+} in the lattice of titania can induce sub-conduction band states, while at higher Zr concentrations TiO_2-ZrO_2 heterojunction is commonly formed. In this case defect states at the interface can be formed. If the Zr at this interface (defects) is present as ionic Zr^{4+} species electrons deriving from TiO_2 can flow to such species with redox potential at approximately -1.45 V vs NHE level and travel further to O_2, thereby reducing it (inset in the Figure S4). The defect formation at the interface is not confirmed in our case and seems unlikely to be present in high enough number to contribute to increased activity under UV light; hence, transfer of e^- to such species and further O_2 reduction seems unlikely and is not confirmed experimentally through higher photocatalytic activity.

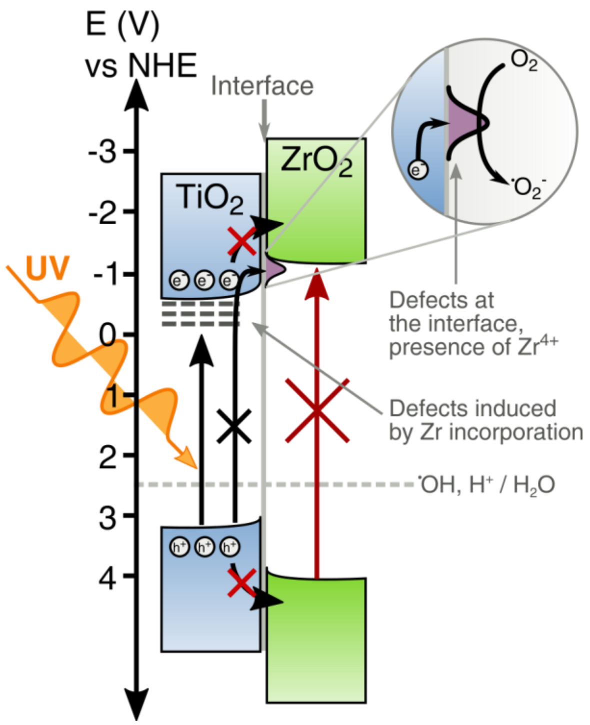

Figure S4. Schematic representation of the band structures of TiO_2 and ZrO_2. Note the defects state below the conduction band of TiO_2 which are created with low concentration Zr-doping. At higher doping (≥ 6 mol.%) defects can be created at the interface between the two oxides. Even then the band positions of ZrO_2 seem to be unfavorable for successful charge carriers' separation unless ionized states of Zr have been created (in high enough number) through which e^- can migrate to O_2. Note the transitions not likely to occur are crossed.
